# Soccer’s AI transformation: deep learning’s analysis of soccer’s pandemic research evolution

**DOI:** 10.3389/fpsyg.2023.1244404

**Published:** 2023-10-16

**Authors:** Jea Woog Lee, Sangmin Song, YoungBin Kim, Seung-Bo Park, Doug Hyun Han

**Affiliations:** ^1^Intelligent Information Processing Lab, Chung-Ang University, Seoul, Republic of Korea; ^2^Department of Artificial Intelligence, Chung-Ang University, Seoul, Republic of Korea; ^3^Graduate School of Advanced Imaging Science, Multimedia and Film, Chung-Ang University, Seoul, Republic of Korea; ^4^Graduate School of Sports Medicine, CHA University, Seongnam-si, Republic of Korea; ^5^Department of Psychiatry, Chung Ang University Hospital, Seoul, Republic of Korea

**Keywords:** soccer, football, pandemic, COVID-19, research trend, topic modeling, BERT, data science

## Abstract

**Introduction:**

This paper aims to identify and compare changes in trends and research interests in soccer articles from before and during the COVID-19 pandemic.

**Methods:**

We compared research interests and trends in soccer-related journal articles published before COVID-19 (2018–2020) and during the COVID-19 pandemic (2021–2022) using Bidirectional Encoder Representations from Transformers (BERT) topic modeling.

**Results:**

In both periods, we categorized the social sciences into psychology, sociology, business, and technology, with some interdisciplinary research topics identified, and we identified changes during the COVID-19 pandemic period, including a new approach to home advantage. Furthermore, Sports science and sports medicine had a vast array of subject areas and topics, but some similar themes emerged in both periods and found changes before and during COVID-19. These changes can be broadly categorized into (a) Social Sciences and Technology; (b) Performance training approaches; (c) injury part of body. With training topics being more prominent than match performance during the pandemic; and changes within injuries, with the lower limbs becoming more prominent than the head during the pandemic.

**Conclusion:**

Now that the pandemic has ended, soccer environments and routines have returned to pre-pandemic levels, but the environment that have changed during the pandemic provide an opportunity for researchers and practitioners in the field of soccer to detect post-pandemic changes and identify trends and future directions for research.

## 1. Introduction

Currently, soccer is the sport with the largest participation and population. Furthermore, the repercussions and revenues generated by soccer worldwide not only affect sports but also have social, economic, and even cultural impacts (Jakar and Gerretsen, 2021). Asken and Rabinovici (2021) reported that approximately 270 million people are actively involved in soccer, which is proof of the sport’s enormous sociocultural and economic impact. Given the large number of people involved in soccer, it is unsurprisingly the most widely and heavily studied of all sports. Indeed, academics are conducting research on all ages and levels of soccer players (Svensson et al., 2016; McFadden et al., 2022; Beavan et al., 2023). Soccer-related research is published in most academic disciplines, including sports sociology, sports science, computer science, engineering, and medicine, with a strong interdisciplinary approach (Williams et al., 2020).

However, on March 11, 2020, the World Health Organization (WHO) declared the highest pandemic alert for COVID-19, specifically categorizing it as a “pandemic” (Wu et al., 2021) and warning that the global spread of COVID-19 was not just a public health crisis but also one with political, economic, social, and cultural implications (Lopez-Bravo et al., 2020). The sports sector was similarly affected by the pandemic. While the responses of national sports leagues varied, international leagues saw a complete suspension of sporting events at all levels (Sivevska and Popeska, 2020). Throughout the pandemic, professional sports leagues had limited or banned spectator attendance; major events like the Olympics and various sports leagues canceled or postponed (Elkhouly et al., 2022), and soccer is no exception.

The COVID-19 pandemic has majorly impacted professional soccer around the world. In Europe, most leagues temporarily suspended play in 2020–2021 to help contain the spread of the virus (Mazza et al., 2022). For a time, players were only allowed to train at home to reduce the risk of infection (Marshall et al., 2022). Team training varied from country to country, league to league, and club to club, but it was conducted carefully and with many controls in place. The COVID-19 pandemic has impacted soccer in a unique and unprecedented way, and this unusual phenomenon has led to a subtle shift in research topics during the pandemic, with some researchers addressing topics related to the pandemic itself.

For example, since the temporary lockdown, leagues have had to play more games than usual in a short period of time to finish on time, and there have been reports of a noticeable increase in player variability and injuries due to long breaks and a lack of training (Seshadri et al., 2021). Studies of possible performance declines during COVID-19’s disruption of matches and soccer-related training have shown declines in sprint and jump tests, which may be related to a reduction in soccer-related (group) training during lockdown (Pierros and Spyrou, 2023). Several studies have examined actual performance and injury rates before and after COVID-19 disruptions in matches and training in several European professional men’s soccer leagues (Thron et al., 2021, 2022). However, it is difficult to determine how specifically and evenly these studies can be applied to the broader field of soccer research. Moreover, overall research trends may have been affected by the COVID-19 pandemic without directly referring to it. This study constitutes another effort to discover and understand these phenomena.

A careful examination of the arguments so far suggests that the COVID-19 pandemic has caused many changes in the soccer landscape and that soccer-related research has also been affected. This observation implies that we can detect some changes in soccer research before and after the pandemic, but we need specific solutions to analyze them more objectively and quantitatively. The development of soccer is growing quantitatively and qualitatively with an exponential increase in research and a multidisciplinary approach. A significant amount of research and empirical reporting is published in academic articles, but extracting this extensive and heterogeneous of knowledge is a complex task. While analyses such as Delphi and meta-analyses are essential, they do not fully capture the scale, scope, and complexity of soccer research. Therefore, it is necessary to consider data science-based approaches to organize the domain knowledge of a large and specific field (Lyu and Costas, 2022). Keyword-driven subject area segmentation with natural language processing can reveal how the vast complexity of knowledge that makes up sports literature is related (Bruner et al., 2010). This rationale can be usefully applied to gain insight into the enormity of soccer research and the various research considerations that have arisen before and after the COVID 19 pandemic. In this study, we used a topic modeling algorithm called BERTopic (Bidirectional Encoder Representations from Transformers Topic) (Grootendorst, 2020), a topic modeling technique that leverages transformers (Vaswani et al., 2017) and class-based term frequency–inverse document frequency (c-TF–IDF) to identify well-studied research areas in the COVID-19 literature related to soccer.

Using topic modeling to study research trends can provide useful information along with new ideas for future research (Lee et al., 2021, 2022). These results can be used to form a body of knowledge by quantitatively analyzing a large number of papers and qualitatively interpreting the discovered knowledge structure to find certain patterns in scattered data (Miyata et al., 2020). The probability distribution of words in a document has no intuitive meaning, but researchers can interpret the meaning of a particular topic and extract insights so that the extracted topic can be used as important information representing the document (Kim et al., 2022). In addition, because multiple related papers are used for analysis, it is possible to discover the relationships between key research topics and various subtopics in the academic field and intuitively explore the knowledge structure by visualizing research results (Park, 2020).

In this study, we use topic modeling with journal articles to analyze the knowledge structure of soccer-related research published before (2019–2020) and during (2021–2022) the COVID-19 pandemic. By comparing the content of the research agendas for the two periods, we aim to determine if there are differences in academic and research interests in soccer before and during the COVID-19 pandemic. In other words, we contrast the trends in soccer-related research topics during the two periods to provide insights into changes, future predictions, and implications.

Therefore, this study aims to comprehensively identifying and comparing the changes in research interests, topics, and trends from before COVID-19 to during the COVID-19 pandemic.

To address the research aims, the research question was set as follows.

Q1. What are the topic areas of soccer research before, during, and after the COVID-19 pandemic?

Q2. What are the topical areas of soccer research organized from the extracted topics?

Q3. How have the topics and topic areas of soccer research changed before, during the COVID 19 pandemic?

## 2. Background

### 2.1. Document embedding

In topic modeling, document embedding is used to convert the raw text of a document into a mathematical representation that can be compared semantically, i.e., documents containing the same topic are semantically similar. This technique is commonly used in topic modeling, which is the process of identifying latent topics in a corpus of text documents. To perform the embedding step in this study, we used BERTopic’s Sentence-BERT (SBERT) (Reimers and Gurevych, 2019), which is a method for encoding sentences or short texts into dense vector representations using Bidirectional Encoder Representations from Transformers (BERT). These vectors capture the meaning and context of the sentence or text, making them useful for various natural language processing (NLP) tasks, including document similarity and clustering.

### 2.2. Term frequency–inverse document frequency

One weighting method for representing text is term frequency–inverse document frequency (TF–IDF). The basic idea is to create a vector for each document in the corpus, where each element of the vector represents a specific feature or term of the document. The value of the element is typically a weight that reflects the importance of the feature in the document. A TF–IDF value is determined according to the relative frequency of a word in a specific document based on its frequency in the document and its rarity across all documents in the corpus. The TF–IDF is given by Equation (1) below, where the weight of the word i in the document j is $W_{ij}$ , *N* is the number of documents, $tf_{ij}$ is the frequency of the word i in the document j , and $df_{i}$ is the number of documents that contain the word i (Zhang et al., 2011).


(1) $$W_{ij}=tf_{ij}*\log\frac{N}{df_{i}}$$

### 2.3. Topic modeling with BERT

Topic modeling is an analytical, unsupervised learning model that discovers topics in a corpus. In this sense, a topic can be defined as a repeated pattern of terms (Gunjan et al., 2021). Recently, deep-learning-based models such as BERT have shown exceptional performance in various NLP tasks, including document embedding and topic modeling called BERTopic (Grootendorst, 2020). BERT is a pre-trained language model that uses a transformer architecture to encode text into dense vector representations. These vectors capture the context and meaning of the text, making them useful for many downstream NLP tasks, including topic modeling. The algorithm uses three primary phases to produce a topic’s distribution for a set of documents. First, it creates sentence embedding. Second, it creates clusters of semantically similar sentences. The last step includes creating topic representation with c-TF–IDF. We elaborate on each step in the Methods section.

Initially, BERTopic works by generating dense vector representations of each document in a corpus using BERT or SBERT; we take the sentence embedding from our documents. These embeddings, however, are primarily used to cluster semantically similar documents and are not directly used in generating the topics.

Primarily, the BERTopic technique utilizes two algorithms: uniform manifold approximation and projection (UMAP) and hierarchical density-based spatial clustering of applications with noise (HDBSCAN) (Campello et al., 2013). UMAP reduces the dimensionality of the document embeddings before clustering (McInnes et al., 2018). Specifically, UMAP is a powerful dimensionality reduction technique that preserves the original structure into a low-dimensional structure, facilitating more efficient and effective clustering.

The second algorithm, HDBSCAN, is a powerful clustering algorithm that can automatically determine the number of clusters in the data, making it a valuable tool for unsupervised text analysis. Additionally, HDBSCAN can handle noise and outliers, which is particularly important when dealing with large and complex datasets. HDBSCAN provides a robust and efficient method for unsupervised topic modeling in social science research. By leveraging advanced NLP techniques and clustering algorithms, BERTopic can capture the nuances and subtleties of natural language and produce highly interpretable topics that can provide deep insights into the underlying structure and meaning of textual data.

The standard TF–IDF equation is used to calculate the importance of each term within each topic rather than across the entire corpus. In contrast, c-TF–IDF defines the importance of a word within a class (topic) (Grootendorst, 2020) and treats all documents in a single class as a single document. Equation (2) finds the c-TF–IDF of each word, where $W_{ic}$ is the weight of word i in class c . The frequency of word i in class *c* is $tf_{ic}$ . *A* is the average number of words per class, and $f_{i}$ is the frequency of word i across all classes.


(2) $$W_{ic}=tf_{ic}*\log\left(1+\frac{A}{f_{i}}\right)$$

Finally, by calculating the c-TF–IDF score for each term within each topic, BERTopic can identify the most important terms for each topic rather than across the entire corpus. These important terms can then be used to label and interpret the resulting topics, providing a more accurate and specific representation of the underlying themes and trends within the data.

## 3. Methods

### 3.1. Data collection

Soccer studies before and during the COVID-19 pandemic were collected by searching the Web of Science (WOS). Articles were collected from the following indexes: Science Citation Index, Science Citation Index Expanded, Social Sciences Citation Index, and Arts & Humanities Citation Index. Keywords were used in the searches, including “soccer” and “football.” The research data from before COVID-19 included 3,956 studies from 2019 to July 2020, and the research data from the period during the COVID-19 pandemic included 3,423 studies from 2021 to July 2022. Irrelevant articles were excluded from the collected data.

**Figure 1:**
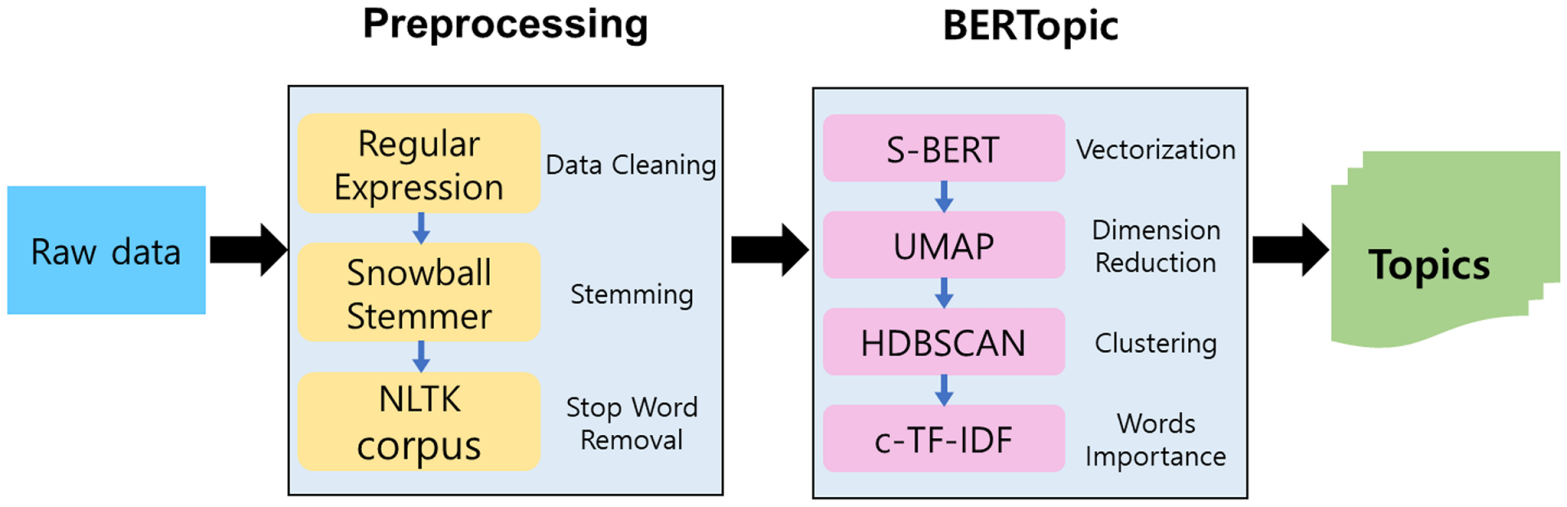
Methodology overview. Research overflow consists of data collection, pre-processing, topic modeling using BERT, and topic analysis.

First, we excluded papers regarding “football” that focused on the National Football League (NFL) in the United States and the Australian Football League (AFL) in Australia. However, articles that presented a comparative approach to soccer were allowed. Articles that were not journal articles (news articles, letters to the editor, research reports, conference proceedings, books, etc.), did not have an abstract, or were written in a language other than English were excluded. The research flow and procedures for this study are shown in Figure 1.

### 3.2. Data pre-processing and topic modeling

This paper proposes a two-step approach for topic modeling in soccer data. The first step involves pre-processing the data to extract relevant information and enhance the original data representation, while the second step uses BERTopic to generate topics that preserve the semantics of the original data representation. The BERTopic algorithm is based on word-embedding models and is capable of identifying relevant topics in the data (Grootendorst, 2020). Specifically, we used a BERT-based embedding model to extract meaningful topics from soccer research data obtained from the WOS. The pre-processing step included data cleaning, tokenization, stemming, and stop-word removal. The following procedure provides an overview of the methodology used in this paper.

1. Data Cleaning: We extracted only English text from the research data using regular expressions to minimize noise in the dataset that might impact topic extraction. We converted all textual contents within the dataset into lowercase to ensure consistency.
2. Stemming: We applied the Snowball Stemmer technique to reduce the word variation in the data and reduce the number of unique tokens in the dataset (Gurcan and Cagiltay, 2023). The remaining words in the dataset were reduced to their stems (e.g., the words “plays,” “playing,” and “played” were all represented as “play”).
3. Stop-Word Removal: We removed common stop words such as “the,” “and,” and “in,” which do not carry significant meaning. Likewise, we filtered out generic words (e.g., “literature,” “paper,” and “study”) that were frequently observed in the articles but did not contribute to the creation of semantically consistent topics. To edit the stop-word list, we utilized NetCorp’s library to modify the stop words. In this way, we ensured that only the words with a single meaning were included in the dataset.
4. Vectorization: Next, we converted the pre-processed text into a numerical format that can be used for analysis, which is embedding. Each document was embedded using the implemented BERT model. We used SBERT to manufacture high-quality contextual word and sentence vector representations.
5. Topic Modeling: Finally, we used BERTopic to perform topic modeling on the pre-processed and vectorized soccer data. BERTopic is a state-of-the-art topic modeling algorithm that utilizes transformer-based language models such as BERT or Sentence-BERT to generate document embeddings, which are then clustered using hierarchical clustering algorithms such as HDBSCAN. This process results in a set of topics, each with a list of important terms and their corresponding weights, which can be interpreted and labeled by the researcher.

In summary, the methodology used in this study consisted of data cleaning, stemming, stop-word removal, vectorization using BERT, and topic modeling using BERTopic. This method enabled us to perform topic modeling on the soccer data systematically and efficiently, while also accounting for potential noise and variations in the data. We implemented the pre-processing steps because we believed they would be beneficial for reducing noise and variation in the data while also having a negligible impact on the semantics of the soccer data. The resulting topics and insights can be used to inform decision-making and analysis in the field of sports and medicine.

As a result of the above method, 47 topics were generated in 8 Topic Areas from the pre-COVID-19 period, and 51 topics were generated in 8 Topic Areas from the period during the COVID-19 pandemic. Clusters of keywords with unclear identities were deleted from the generated topics (Gurcan and Cagiltay, 2022). Therefore, 1 topic was deleted from the pre-COVID-19 pandemic period and 5 topics were deleted from the period during the COVID-19 pandemic.

From the topic modeling results, we first examined the topic areas and keyword clusters of soccer-related articles published before the COVID-19 pandemic; second, we examined the topic areas and keyword clusters of soccer-related articles published during the COVID-19 pandemic. Then, we defined the research topics using the topic areas and clusters mapping the research areas in the two periods and deduced the changes in topic approaches and future directions through comparison.

## 4. Results

### 4.1. Soccer research trends: 2018–2020

The soccer research Topic Areas for the pre-COVID-19 period (2018–2020) and the keyword composition for each Topic Area are shown below. The results of topic modeling for soccer research in the pre-COVID-19 period are shown in Table 1.

**Table 1:** Topic modeling results—soccer research before COVID-19.

Topic Area-1 Social Science-1: Psychology, Sociology, and Business	**Topic 7**	**Topic 10**	**Topic 15**	**Topic 24**	**Topic 37**	**Topic 38**	**Topic 41**	**Topic 46**
	Coach	rae	Parents	Psychology	Reference	Mental	Market	Talent
	Leadership	Age	Youth	Motivation	Decision	Health	Value	Develop
	Style	Born	Children	Burnout	Research	Mindmax	Club	Identify
	Team	Player	Sport	Need	Make	Social	League	Football
	Efficacy	Talent	Practice	Climate	var	Men	Foreign	Financial
Topic Area-2 Social Science-2: Psychology and Socialcultral	**Topic 0**	**Topic 8**	**Topic 22**	**Topic 23**	**Topic 29**	–	–	–
	Fan	Market	Women	Club	Fan	–	–	–
	Football	Attend	Gender	Financial	Brand	–	–	–
	Culture	Uncertainty	Sport	Sport	Sponsor	–	–	–
	Article	League	Football	Football	Sponsorship	–	–	–
	Politic	Stadium	Identify	League	Loyalty	–	–	–
Topic Area-3 Sport Science and Technology	**Topic 5**	**Topic 9**	**Topic 12**	**Topic 40**	**Topic 45**	–	–	–
	Goal	Model	Robot	Wind	Technique	–	–	–
	Team	Predict	Algorithm	Stadium	Revenue	–	–	–
	Possess	Network	Propose	Temperature	Import	–	–	–
	Shot	Pass	System	Roof	Match	–	–	–
	Score	Team	Image	Degree	Variable	–	–	–
Topic Area-4 Sport Medicine-1: Injury	**Topic 1**	**Topic 3**	**Topic 16**	**Topic 19**	**Topic 20**	**Topic 25**	**Topic 26**	–
	Knee	Injury	Cardiac	nfl	Groin	Head	Intake	–
	Degree	Incidence	Cardiovascular	Combination	hip	Impact	Nutrition	–
	Strength	Rate	Ventricular	Injury	Pain	Acceler	Energy	–
	Hamstring	Muscle	Myocardi	Tear	Injury	Magnitude	Diet	–
	Muscle	School	Left	Labral	Adductor	Linear	Carbohydrate	–
	**Topic 26**	**Topic 28**	**Topic 32**	**Topic 36**	**Topic 42**	**Topic 44**	–	–
	Intake	Injury	Turf	Bone	Head	Pelvic	–	–
	Nutrition	Risk	Artificial	bmd	Header	Injury	–	–
	Energy	ci	Surface	bmc	Headcount	Factor	–	–
	Diet	Factor	Infiltration	Female	Exposure	Hamstring	–	–
	Carbohydrate	Season	Nature	Male	Impact	lbp	–	–
Topic Area-5 Sport Medicine-2: Brain Injury	**Topic 11**	**Topic 43**	–	–	–	–	–	–
	Brain	Symptom	–	–	–	–	–	–
	Tau	Cognit	–	–	–	–	–	–
	cte	Concuss	–	–	–	–	–	–
	Traumat	adhd	–	–	–	–	–	–
	Head	History	–	–	–	–	–	–
Topic Area-6 Sport Science-1: Performance	**Topic 17**	**Topic 39**	**Topic 47**	–	–	–	–	–
	Body	Strength	COD	–	–	–	–	–
	Fat	nh	rag	–	–	–	–	–
	Equate	Sprint	fs	–	–	–	–	–
	Composite	Maturity	Test	–	–	–	–	–
	Mass	Jump	Direct	–	–	–	–	–
Topic Area-7 Sport Science-2: Physiology and Nutrition in Performance	**Topic 4**	**Topic 6**	**Topic 13**	**Topic 21**	**Topic 18**	**Topic 31**	**Topic 34**	**Topic 35**
	Vitamin	hr	Distance	Cortisol	Load	gps	Pitch	Sleep
	Supplement	Heart	Speed	Testosterone	Week	System	Format	Night
	Blood	Min	Match	Salivari	Train	Track	vs	Quality
	Exercise	Rate	Demand	Mood	Workload	gen	Size	nap
	Level	hrv	Accelerate	Sweat	Season	Distance	Distance	Even
Topic Area-8 Sport Science-3: Visual Skill in Performance	**Topic 2**	**Topic 14**	**Topic 27**	**Topic 30**	–	–	–	–
	Jump	Kick	Visual	Creative	–	–	–	–
	Train	Ball	Saccad	Task	–	–	–	–
	Sprint	Foot	Screen	Mental	–	–	–	–
	Group	Velocity	Eye	Control	–	–	–	–
	Improve	Leg	Task	Condition	–	–	–	–

Topic Area-1 was labeled “Social Sciences-1: Psychology & Sociology” related to soccer and includes the following Topics. Topics 7, 15, and 24 were identified as psychological topic areas. Specifically, Topic 7 was clustered with keywords about team or athlete efficacy based on the coach’s leadership style. Topic 15 was clustered with keywords that explored various variables related to parents that affect youth and children soccer players. Topic 24 was clustered from straightforward keywords like psychology to keywords about soccer player motivation and burnout. Topic 38 was clustered around validated interventions for soccer players’ mental health, with keywords ranging from players’ social skills to mental-healthcare-related smartphone applications (mindmax, MindMax LLC, Duxbury).

On the other hand, Topics 10 and 46 were found to be clustered around soccer-related sociology topics. Topic 10 clustered keywords related to identifying the social and environmental determinants (age, born, talent, etc.) of the relative age effect (REA) on soccer players. Topic 46 clustered research topics related to soccer player talent identification and development; we also found one soccer business and one technology topic in this Topic Area. Topic 37 was clustered with keywords related to identifying changes in soccer game decision-making following the introduction of video assistant referee (VAR) systems, and Topic 41 was clustered with keywords related to exploring market value determinants (such as foreign players) of each club in professional soccer leagues.

Next, Topic Area-2 has the domain name “Social Sciences-2: Business & Sociocultural” as related to soccer and includes the following Topics. Topics 8, 23, and 29 were identified as detailed research topics related to the soccer business. Topic 8 was clustered with keywords exploring the determinants of stadium attendance in soccer leagues. Topic 23 was clustered with keywords related to evaluating the financial performance of a soccer league or club. Topic 29 was clustered with keywords representing topics such as sponsor, sponsorship, and loyalty that affect the formation of a fan’s brand image of a club or league. Topics 0 and 22 were identified as sociocultural themes. Topic 0 was clustered into thematic keywords dealing with soccer fans’ culture and political ideology, and Topic 22 was clustered under topic keywords dealing with sociological issues, including the perceptions, stereotypes, gender roles, and identities of female soccer players.

Topic Area-3 was labeled “Sport Science & Technology” as related to soccer and includes the following Topics. Topic 5 was clustered with keywords related to data analysis, which is related to team performance topics such as ball possession, shots, and score. Topic 9 was clustered with keywords representing research related to power analysis using a team’s pass network and prediction model. Topic 12 was clustered with keywords related to research on the structural response of stadium roofs to wind, temperature, etc., in terms of structural engineering. Topic 12 was clustered with keywords that propose, apply, and validate algorithms for image-processing tracking systems that recognize opposing robots and balls for soccer robot functions. Topic 45 was clustered with keywords related to the technique of exploring the determinants of how to import variables that generate revenue from soccer matches.

Topic Area-4 has the domain name “Sport Medicine-1: - Injury” as related to soccer and includes the following Topics. First, Topics 1, 20, and 44 were clustered with research keywords related to injuries in the “lower limb” area, such as “knee,” “hamstring,” “pelvic,” “groin,” and “adductor,” and dealt with the factors and symptoms of such injuries. In addition, “nfl” appeared in Topic 19, which is a keyword for the American NFL, and was identified as a topic generated in the process of comparing injuries according to the sport characteristics of American football and soccer.

Research on head-related injuries was also identified. Topic 25 was clustered under the subject keywords “acceleration” and “impact magnitude,” which explored acceleration and impact magnitude depending on the head region studied. Topic 42 was clustered under topic keywords related to monitoring impact exposure and measurement based on athlete recall through Headcounter, an athlete self-report questionnaire on headers. Topic 36 was clustered with keywords representing topics that explored differences in bone mineral content and bone mineral density according to demographic characteristics, such as sport and gender, and the types of injuries they cause. Topic 16 consists of keyword clusters representing topics related to myocardium injuries in soccer players.

Topics 3, 26, 28, and 32 consist of topics related to lifestyle and environmental factors that lead to injury. Topics 3 and 28 were clustered with topic keywords that explore the relationship between injury risk, injury-triggering factors, and injury incidence in elite soccer players and school elite players. Topic 26 was clustered with keywords describing topics related to energy expenditure, nutrient intake, and weight control in soccer players and their relationship to performance and injury. Topic 32 was clustered with keywords describing topics related to infiltration for safe and effective turf surface stability, including topics comparing injury rates and player fatigue on natural and artificial turf.

Topic Area-5 was labeled “Sport Medicine-2: Brain Injury” as related to soccer and includes the following Topics. Topic 11 is centered on chronic traumatic encephalopathy in soccer players and is clustered with topic keywords covering dementia, sequelae such as Alzheimer’s, and delaying and preventing tau protein through immunization. Topic 43 was clustered under the topic keyword “concussion,” which explores the relationship between soccer players’ cognitive abilities, history of ADHD, etc.

Topic Area-6 was labeled “Sport Science-1: Exercise Performance” as related to soccer. In this Topic Area, Topic 17 was clustered into topic keywords that explored the relationship between soccer performance and complex body composition, such as player weight, body fat percentage, and body mass index (BMI), from various perspectives. Topic 39 was clustered with topic keywords that explored the relationship between maturity and soccer performance from various perspectives, including players’ basic physical fitness factors such as strength, jump, and sprint. Topic 47 was clustered with keywords that addressed topics related to soccer players’ athletic performance, such as change of direction (COD) and reactive agility.

Topic Area-7 was labeled “Sport Science-2: Biology & Performance & Nutrition” and includes the following Topics. Topic 4 was clustered with keywords related to nutrition and performance, including nutritional intake, eating habits, and blood measurements for validation. Topic 6 was clustered with keywords representing research related to the role of the heart, such as heart rate and cardiac output in athletes’ performance during competition and training. Topic 13 was clustered with keywords representing the demands of athletic performance for effective performance in a match, such as distance, speed, and acceleration. Topic 18 was clustered with keywords that explored the relationship between an athlete’s training load during the season and their performance. Topic 21 was clustered into topics that explored biological studies such as sweat production, cortisol and testosterone secretion, and mood during athletic performance such as competition and training. Topic 31 is clustered with keywords that refer to performance measurement research topics using digital devices such as GPS, such as distance markers and player tracking, which are used to measure performance in athletic and training situations. Topic 34 is clustered with keywords that explore the performance and physiological responses of youth or adult players to soccer field dimensions, such as exercise volume and exercise characteristics. Topic 35 was clustered with keywords related to analyzing sleep habits such as insomnia in soccer players or exploring the effects of interventions such as effective sleep and NAP to improve performance.

Topic Area-8 was labeled “Sport Science-3: Visual Skill in Performance.” In this Topic Area, Topic 30 was clustered under the following research topic keywords: “cognitive control,” “cognitive state,” and “psyche in soccer-specific creative expression.” Topic 27 was clustered under the topic keywords exploring the level of soccer players’ saccade eye movements and its relevance to tasks and performance in different settings and interventions. Topic 14 was clustered under topic keywords related to factors that contribute to an effective kick, such as kick speed, angular velocity, and foot (right or left) use. Topic 2 was clustered under research keywords related to jump training interventions for various performance enhancements, including sprinting, one of the most important motor skills in soccer. The pre-COVID (2018–2020) Topic Areas and Topics are shown in Table 1.

### 4.2. Soccer research trends: 2021–2022

The soccer research Topic Areas for the period during the COVID-19 pandemic (2021–2022) and each Topic’s keyword organization are as follows, and the topic modeling results for soccer research from this period are shown in Table 2.

**Table 2:** Topic modeling results—soccer research during COVID-19.

Topic Area-1 Social Science-1: Business and Sociology	**Topic 0**	**Topic 8**	**Topic 1**	**Topic 31**	**Topic 37**	–	–	–
	Team	Club	Social	Club	Home	–	–	–
	Match	Transfer	crm	Brand	Advantage	–	–	–
	Play	Financial	Intern	Vote	Crowd	–	–	–
	Pass	Market	csr	Climate	Covid	–	–	–
	Game	European	Response	Fan	ha	–	–	–
Topic Area-2 Social Science-2: Psychology and Socialcultral	**Topic 3**	**Topic 5**	**Topic 9**	**Topic 11**	**Topic 14**	**Topic 23**	**Topic 28**	**Topic 38**
	Coach	Politic	Cognitive	Fan	Women	Self	Mental	Fan
	Leadership	Nation	Visual	Fandom	Gender	Psychology	Anxiety	Sexual
	Parent	Identity	Task	Media	Men	Perfection	Health	Homosexual
	Question	Culture	Skill	Social	Coverage	Efficacy	Depress	Men
	Education	Media	Eye	Club	Nation	Cope	Covid	Masculine
Topic Area-3 Sport Science and Technology	**Topic 15**	**Topic 17**	–	–	–	–	–	–
	Video	Robot	–	–	–	–	–	–
	Track	Learn	–	–	–	–	–	–
	Detect	Agent	–	–	–	–	–	–
	AI	Humanoid	–	–	–	–	–	–
	Target	Algorithm	–	–	–	–	–	–
Topic Area-4 Sport Medicine-1: Lower Limb Injury	**Topic 3**	**Topic 26**	**Topic 47**	–	–	–	–	–
	algorithm	cai	groin	–	–	–	–	–
	Strength	Ankle	Pain	–	–	–	–	–
	Torque	Land	hip	–	–	–	–	–
	Flexor	Balance	hago	–	–	–	–	–
	Limb	Group	Subscale	–	–	–	–	–
Topic Area-5 Sport Medicine and Science-1	**Topic 20**	**Topic 21**	**Topic 24**	**Topic 30**	**Topic 44**	–	–	–
	Nutrit	Infect	Lockdown	Cardiac	Menstrual	–	–	–
	Intake	Sars	Covid	ecg	Cycle	–	–	–
	Dietari	cov	Period	lv	Breastfeed	–	–	–
	Knowledge	Covid	Pandemic	Ventricular	hc	–	–	–
	Energy	pcr	Confidence	Coronaria	Hold	–	–	–
Topic Area-6 Sport Medicine and Science-2	**Topic 4**	**Topic 6**	**Topic 10**	**Topic 12**	**Topic 22**	**Topic 27**	**Topic 29**	**Topic 34**
	Jump	Supplement	Starter	aerob	ssg	Sleep	Load	Neuromuscular
	Sprint	Vitamin	Season	Test	ssgs	Quality	Train	Response
	Train	Blood	Week	fit	Sprint	Night	srpe	Matur
	Performance	Acid	md	vo	Side	Duate	External	Train
	Group	Metabolism	Load	Oxygen	Train	Insomnias	Session	Post
	**Topic 36**	**Topic 39**	**Topic 41**	**Topic 42**	**Topic 45**	**Topic 46**	**Topic 49**	**Topic 51**
	Week	Kick	Cortisol	gps	Bone	Goalkeep	Match	Stretch
	Load	Ball	Recovery	Track	bmd	Penalty	Peak	Soleus
	Workload	Foot	Hormone	Reliability	Miner	Kick	Distance	Strength
	Injury	Velocity	ck	Polar	Density	Shot	ft	Squat
	Chronic	Pelvis	cwi	Distance	Age	Kicker	Demand	Square
Topic Area-7 Sport Medicine-3: Injury	**Topic 1**	**Topic 2**	**Topic 19**	**Topic 25**	**Topic 32**	**Topic 43**	–	–
	Head	Injury	mri	acl	Turf	Program	–	–
	Impact	Incident	Image	Reconstruct	Grass	Injury	–	–
	Exposure	Season	Injury	Injury	Artificial	Prevent	–	–
	Brain	rtp	Patient	Knee	Rubber	Hamstring	–	–
	Concuss	Play	Lesion	Ligament	Nature	Intervention	–	–
Topic Area-8 Sport Science: Performance	**Topic 13**	**Topic 16**	–	–	–	–	–	–
	rae	Anthropometric	–	–	–	–	–	–
	Agent	Age	–	–	–	–	–	–
	Talent	Maturity	–	–	–	–	–	–
	Maturity	Test	–	–	–	–	–	–
	Select	Sprint	–	–	–	–	–	–

Topic Area-1 is a soccer-related domain named “Social Sciences-1: Business & Sociology with Covid-19” and includes the following Topics. Topic 1 is clustered with research topic keywords related to Corporate Social Responsibility (CSR) and Customer Relationship Management (CRM), which are common in the sports business field. Topic 8 clustered together topics related to player transfers in European soccer leagues and exploring league and club financial factors. Topic 31 was clustered under the keyword “socio-business,” which refers to soccer club branding; franchise culture, such as fan and club communication; and socio-business research that explores the social identity of clubs for business and, in the process, community club activities and fan identities. Topic 37 is clustered under the keyword “socio-business,” which refers to studies exploring the effects of home advantage, focusing on unattended professional soccer matches in the context of COVID-19. Topic 0 was clustered with topic keywords that explored the socio-scientific factors affecting performance from various perspectives, including match-play analyses of players during matches.

Topic Area-2 was labeled “Social Science-2: Psychology & Sociocultural with COVID-19” as related to soccer and includes the following Topics. Topic 23 was clustered into keywords that represented general psychological research topics such as soccer players’ perfectionism, self-efficacy, and coping strategies for various negative psychological factors. Topic 3 was clustered under keywords of research topics that explored coach leadership, parenting styles, and educational environments as they impact soccer player development. Topic 28 was clustered with keywords representing studies that explored specific causal relationships between mental health issues experienced by soccer players during COVID-19, such as anxiety and depression. Topic 38 was clustered under the keyword “sociological issues in soccer related to sex and gender,” including gender roles in men’s and women’s soccer, LGBTQ fan attitudes and their impact on professional soccer as a whole, and LGBTQ players. Topic 14 was clustered under the keyword “sociological issues in soccer,” referring to topics on issues related to discrimination against female athletes in sports from a sociological perspective, using women’s soccer as a case study. Topic 11 was clustered under the keywords “social media” and “social communities” and keywords that related to other social activity opportunities for clubs to build and maintain a fanbase, as well as methods and effects of club–fan interactions. Topic 5 was clustered under the keyword “political and social issues,” including national branding through soccer, the media’s role in image-making, and soccer fans’ national identity. Topic 9 was identical to one of the topics in the “Sport Science-3: Visual Skill” Topic Area from the pre-COVID-19 period. This can be attributed to the inclusion of the psychology keyword “cognitive” in this Topic Area, as the “Sport Science-3: Visual Skill” Topic Area was not formed in the COVID-19 period.

Topic Area-3 was renamed to the soccer-related domain “Sport Science & Technology.” Topic 15 was clustered under the research topic keywords related to the development and validation of artificial intelligence (AI) algorithms for automatically tracking player movements from soccer videos. Topic 17 shows a similar keyword cluster to Topic 12 in the pre-COVID-19 period and was clustered with topic keywords related to enhancing humanoid functions through machine learning for AI soccer robots.

Topic Area-4 was labeled “Sport Medicine-1: Lower Limb Injury-1,” a topic similar to Lower Limb Injury in Topic Area-4 (“Sport Medicine-1: - Injury”) from the pre-COVID-19 period. Topic 47 was clustered with keywords of research topics related to athlete thresholds for minimizing lower extremity injuries through hip and hip outcome scores. Topic 26 clustered keywords for topics that explored the relationship between chronic ankle instability, which has various causes such as playing soccer and landing after jumps during training. Topic 7 was clustered with topic keywords that explored the relationship between the level of knee muscle extension and injury in soccer players, applying various subjects and external factors.

Topic Area-5 was labeled “Sport Medicine & Science-1” related to soccer and includes the following Topics. Topic 44 was clustered with keywords that explored the physiological phenomena of female soccer players, such as hormonal contraception, pregnancy, menstruation, and breastfeeding, and factors related to athletic activities, such as performance and injury. Topic 20 was clustered with keywords describing topics related to energy expenditure, nutritional intake, and weight control in soccer players, similar to Topic 26 in the pre-COVID-19 period, as well as performance and injury. Topics 21 and 24 can be seen as clusters of topics that address the impact on various soccer stakeholders arising from the fear of infection due to COVID-19 and the medical issues of lockdown. Topic 30 consists of a cluster of keywords describing topics related to myocardium injuries in soccer players, similar to Topic 16 in the pre-COVID-19 period.

Topic Area-6 is soccer-related, and the domain is named “Sport Medicine & Science-2.” Because this Topic Area includes many topics, it was divided into subtopic areas. Topics 6, 12, and 41 have subtopic domains named “Sport Medicine-3: Biology & Nutrition in Performance.” Topic 6 was similar to Topic 4 in the pre-COVID-19 period and was clustered with nutrition and performance-related keywords, including nutrition as a factor in athletic performance, the relationship between diet and immunity, and blood measurements for validation. Topic 12 was clustered with research topics that explored the relationship between physiological measures of aerobic fitness levels and performance ability. Topic 41 was clustered under the keyword “measurement and validation,” which refers to research on hormones, including cortisol, and serum tests for creatine kinase to explore the effects of cold-water immersion on soccer players’ recovery process.

In addition, Topics 27, 39, 42, 46, and 49 include a subtopic domain named “Sport Science-1: Exercise Performance.” In the pre-COVID-19 period, these domains were categorized as independent Topic Areas, but during the COVID-19 period, they were included in Topic Area 6. Topic 27 was clustered with research keywords that explored the relationship between sleep quality and training, performance, and injury in soccer players. Topic 39 was similar to Topic 14 in the pre-COVID-19 period; it clustered topic keywords related to factors that contribute to effective kicking, such as kick speed, angular velocity, and the use of the (right or left) foot. Topic 42 was similar to Topic 31 in the pre-COVID-19 period and clustered around keywords such as “GPS” and “Polar,” which are used as distance markers and measuring instruments in performance research in athletic and training situations. Topic 46 was clustered with research topics that explore goalkeepers’ behavioral characteristics in match situations, either throughout the match or in special situations such as penalty kicks. Topic 49 was similar to Topic 13 in the pre-COVID-19 period and was clustered with keywords that represent the athletic demands of effective performance on the field, such as distance traveled, maximum velocity, and acceleration.

Topics 4, 10, 22, 29, 34, 36, and 51 include a subtopic domain named “Sport Science-2: Performance Training.” Topic 4 was similar to Topic 2 in the pre-COVID-19 period and was clustered with research keywords related to jump training interventions for various performance enhancements, including sprinting, which is one of the most important motor skills in soccer. Topic 10 was similar to the pre-COVID-19 period (2018–2020), clustered with keywords that refer to topics related to players’ training load and performance in and out of season. Topic 22 was clustered with keywords for research exploring the relationship between small-sided games (SSG), a soccer training method, and player performance. Topic 29 was clustered with keywords for research topics that explore the relationship between external loading and athlete session ratings of perceived exertion during training in an attempt to determine effective training volume and training strategies. Topic 34 was clustered under the topic keywords exploring the relationship between training and various maturation and developmental factors, such as neuromuscular and respiratory factors, in athletes. Topic 51 was clustered with topic keywords that explored stretching methods, intensities, methods (squats, etc.), postures, and areas (soleus) for effective lower extremity training.

Topics 36 and 45 included a subtopic domain named “Sport Medicine-4: Injury-2.” Topic 36 was similar to Topic 18 in the pre-COVID-19 period. However, the word “season” was removed and clustered with a slightly different keyword composition, specifically, with topics that explored the relationship between training load and injury, such as “chronic” and “Injury.” Topic 45 also appeared to be similar to Topic 36 in the pre-COVID-19 period; it was clustered with keywords representing topics that explored differences in bone mineral content and bone mineral density according to demographic characteristics such as sport and gender, as well as types of injuries.

Topics 1, 2, 19, 25, 32, and 43 were included in the topic domain “Sport Medicine-5: Injury-3.” Topic 1 appeared similar to the head and brain injury topics in the pre-COVID-19 period but was implied as a single topic. Topic 1 was clustered with topic keywords covering injuries such as concussions due to impact exposure when heading. Topic 2 was clustered with keywords describing two topics, one related to the performance of athletes returning from injury and the other related to the performance of athletes returning after COVID-19. Topic 19 was clustered with keywords describing topics related to the careful measurement and diagnosis of an athlete’s injury using MRI images to verify a precise causal relationship with symptoms. Topic 25 was clustered with keywords describing different research approaches to repetitive injuries to the same and surrounding areas after anterior cruciate ligament reconstruction. Topic 32 had the same number as Topic 32 in the pre-COVID-19 period and was clustered under the keyword “infiltration for safe and effective turf surface stability,” with topics comparing injury rates and player fatigue on natural and artificial turf. Topic 43 was clustered under the keyword “research,” which refers to topics that validated various interventions for the recovery and prevention of hamstring injuries.

Topic Area-8 was labeled “Sport Science-3: Exercise Performance-2.” Topic 13 was clustered under the keyword “relative age effect” (REA), which refers to various research topics approaching REA from a performance perspective. Topic 16 appeared to be similar to Topic 39 in the pre-COVID-19 period and was clustered under the keyword “maturity,” which refers to a variety of research topics that explored the relationship between maturity and soccer performance, including anthropometrics, demographic characteristics such as age, and athletic performance such as running. The Topic Areas and Topics from the period during the COVID-19 pandemic (2021–2022) are shown in Table 2.

### 4.3. Visualization

Visualization was also conducted to ensure that well-defined clusters with interpretable topics were acquired. An intertopic distance map is a visualization technique that shows the similarity between topics represented as the distance between each topic. A topic is a node, and the set between nodes is organized as a topic area. Topics that resulted in similar topic areas overlapped.

Despite the variations in location across the quadrants, the social science topic areas were located close together. The sports science and sports medicine topic areas were also located close to each other, although there was a shift in position in the quadrant. However, during COVID-19, Topic Area-4 was clustered with keywords focusing on movement-related factors associated with lower extremity injuries, which may have distanced it from the sports science and sports medicine areas, including Topic Area-6. While the pre-COVID-19 period is characterized by a clustering of injury-related topics, the period during the COVID-19 pandemic is characterized by an overlap of performance-related topics and a scattering of injury-related topics. Insights into visual changes are derived in the Discussion section.

**Figure 2:**
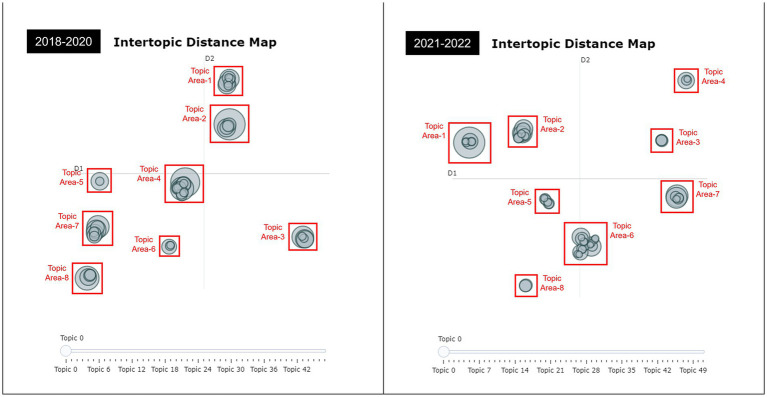
Intertopic distance map of soccer research before and during COVID-19.

**Figure 3:**
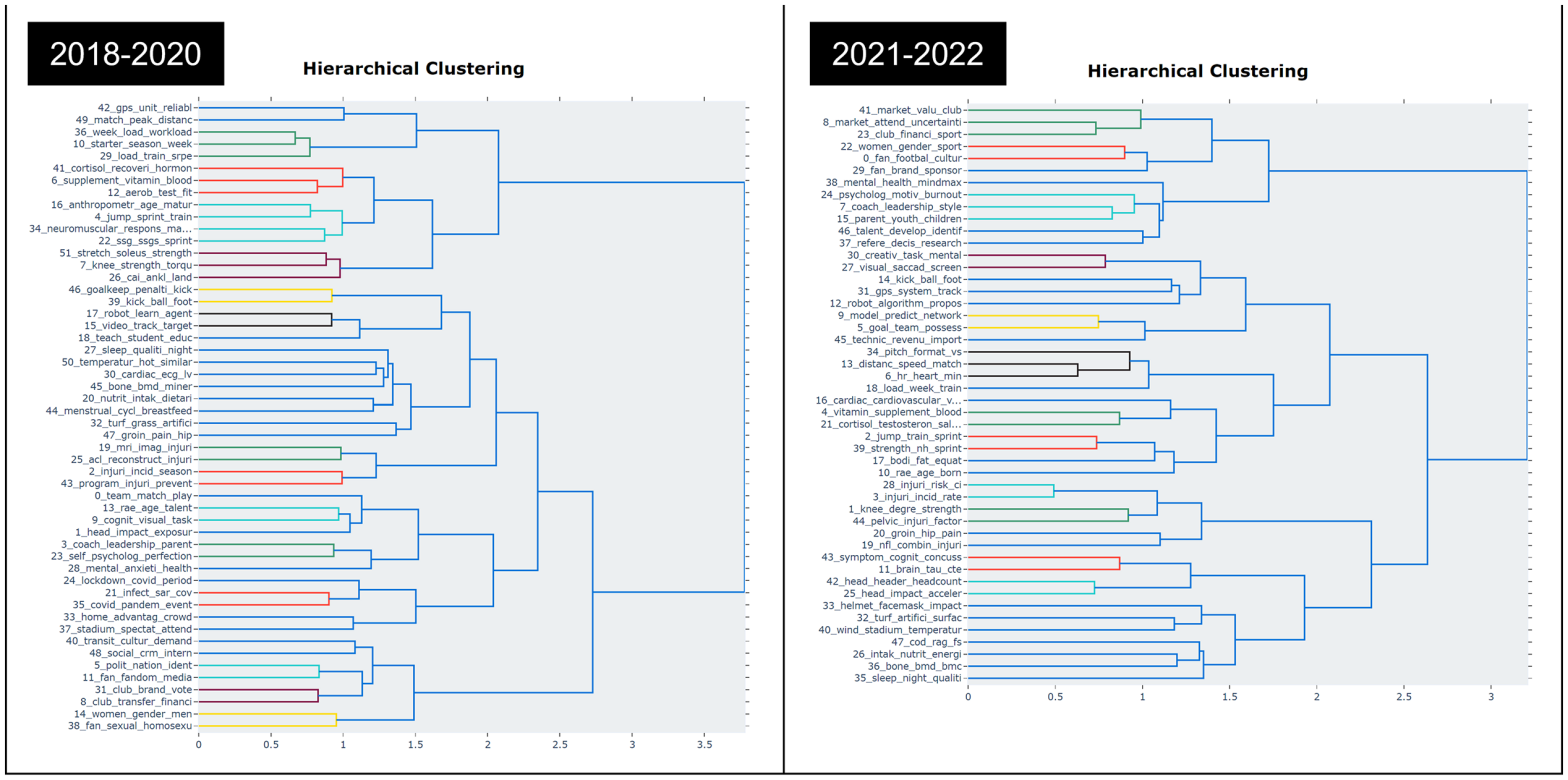
Hierarchical clustering of soccer research before and during COVID-19.

In addition, we utilized hierarchical clustering, a technique in topic modeling to visualize the hierarchical structure of topics, to analyze the topics between 2018–2020 and 2021–2022. We also used a ward-linkage function to perform hierarchical clustering based on the cosine distance matrix between topic embeddings (Ward, 1963). Figure 2 enables us to gain insights into the relationships between topics and identify clusters of related topics. By examining the clusters and their relationships, we can better understand the underlying themes and patterns present in the data. Overall, Figure 3 shows the effectiveness of hierarchical clustering in topic modeling analysis. These findings provide valuable insights for researchers and practitioners in the field and contribute to the growing body of knowledge around topic modeling.

**Figure 4:**
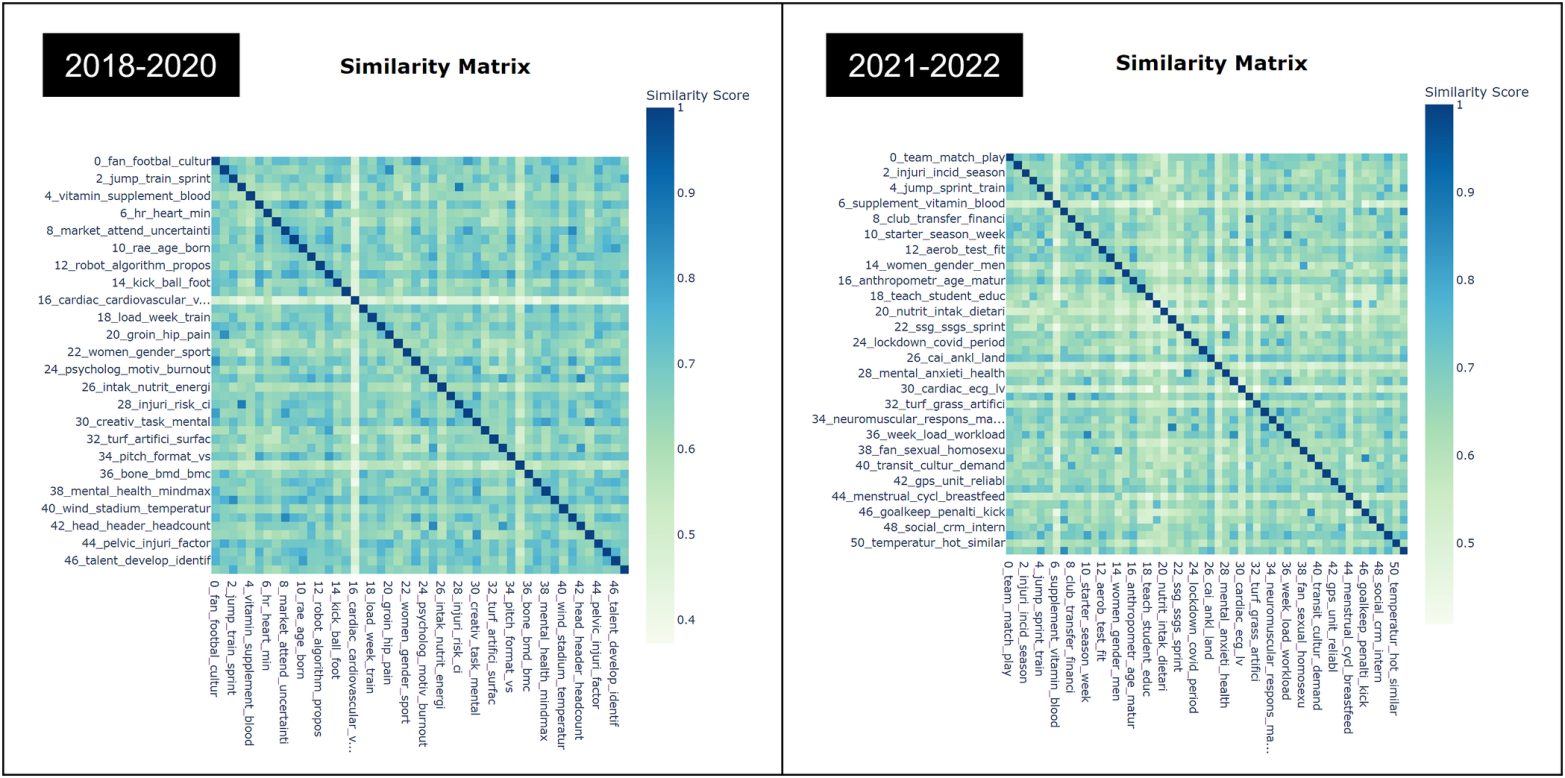
Similarity matrix of soccer research before and during COVID-19.

In addition to the hierarchical clustering, we also utilized a heatmap visualization technique to gain further insights into the similarities between the topics in our paper. A heatmap based on the cosine similarity matrix between topic embeddings was created to show the similarities between topics. Figure 4 provides a clear and intuitive visualization of the relationships between the topics, with darker shades indicating a higher degree of similarity. By examining the heatmap, we were able to identify clusters of similar topics and gain a deeper understanding of the underlying themes and patterns present in the data.

Furthermore, we were able to visually confirm both strong and weak similarities between topics by reviewing and analyzing the heatmap visualization results, which provided valuable insights into the relationships between them. The resulting visualization enabled us to gain a deeper understanding of the underlying patterns and themes present in the data and provided a useful framework for further analysis and interpretation of our results.

## 5. Discussion

### 5.1. Social sciences in soccer

The topic clusters and topic areas established in this study demonstrate the close relationship between the social sciences, sports science, and sports medicine in soccer. Topic areas and topics were separated or merged over time, and keywords were changed, too. Nevertheless, we derived insights by keeping the connections between complex and interrelated topics as tight as possible.

#### 5.1.1. Psychology

In terms of general factors, we found that the research on the psychology of soccer exhibited similar prominent thematic keywords both before and during the COVID-19 pandemic. “Self-efficacy,” “coach leadership,” and “relationship with parents” were common in both periods (Teques et al., 2019; Hong and Jeong, 2020; Schatz et al., 2020; Maurice et al., 2021; Eckardt et al., 2022). During the COVID-19 pandemic, the keywords “motivation” and “burnout” became less prominent, suggesting that they became less salient as pandemic-related research became more important.

Interestingly, the process of exploring positive and negative factors related to mental health was also common in both periods but changed between them. In the pre-COVID-19 period, multiple studies were found that investigated soccer players’ mental health or tested its causal relationship with various intervention variables, including the digital environment, and applied soccer interventions for people with mental health problems (Abbott et al., 2019; Kunrath et al., 2020; Thompson et al., 2020). During the COVID-19 pandemic, a large proportion of the research was inductive, exploring the mental health issues experienced by players in the context of the various restrictions imposed as a result of the pandemic (Jordana et al., 2022; Zhao et al., 2022), suggesting a difference in approach to mental health compared to the pre-pandemic period.

Additionally, studies from the pre-COVID-19 period on soccer players’ visual skills were categorized as “performance” (Song et al., 2019; Bekris et al., 2020). However, during the COVID-19 pandemic, the Topic Area “Performance-Visual Skill” disappeared, and cognitive keywords that are important in visual-skill research emerged, which could be interpreted as a topic area in the field of psychology (Casella et al., 2022; Knollner et al., 2022). Soccer players’ psychological state can be attributed to both self-influences and external/environmental factors (Schinke et al., 2018). Therefore, it is expected that researchers will continue to publish results on the psychological issues experienced by soccer players in the current COVID-19 pandemic situation. In addition, the new phase of soccer players’ experience in the post-COVID-19 environment will be an important issue that may continue to be explored in the field of psychology.

#### 5.1.2. Sociology

The sociological study of soccer was discovered to be more interdisciplinary than general sociological research, with a variety of topic meanings and keywords that were similar in both time periods studied. Nevertheless, soccer studies that addressed sex and gender, such as the perceptions and gender roles of female soccer players and the attitudes and impact of LGBTQ fans, were identified in both periods (Petty and Pope, 2019; Bevan et al., 2021; Kossakowski and Besta, 2022; Mikkonen, 2022). In addition, studies exploring fans’ political behaviors, attitudes, projected national identities, and ideologies in relation to soccer were also deeply sociological (Tamir, 2019; Baltas, 2021; Oonk, 2021; Webber, 2022) and have been noted as thematic issues across both periods.

Although the keywords organized in each topic vary by time period, there were many sociological studies centered on fan culture. In the pre-COVID-19 period, keywords such as “ideology” and “politics” were used to identify fans’ sociocultural behavioral characteristics (Smith and Lord, 2018; Papadima and Photiadis, 2019). During the COVID-19 pandemic, fan-focused sociological research topics expanded compared to the pre-COVID-19 period.

Indeed, the establishment of club–fan relationships and identities conveyed to fans through social activities and promotions, online interactions, community club activities, etc., can be studied, and such studies can be characterized as sociological research toward the ultimate goal of a viable and successful business approach that includes the branding of football clubs (Calabuig et al., 2021; Mora et al., 2021; Weber et al., 2022; Brand et al., 2023; Fenton et al., 2023). In fact, Calloway et al. (2019) and Radmann and Karlen (2022) reported that the most important aspect to consider in a catastrophic crisis such as lockdowns and reduced league operations due to COVID-19 is the relationship with fans. This need was predicted to be the cause of the fan-centered sociological studies conducted during the pandemic.

On the other hand, research topics emerged that could be approached in the unique context of the COVID-19 pandemic. For example, the scholarly soccer literature explored whether there was still a significant home-advantage effect in soccer matches played away from home during the pandemic (McCarrick et al., 2021; Lee et al., 2022). Through this topic, future research directions can be anticipated as areas where comparative approaches can be made considering the current post-COVID-19 situation. Overall, similar to psychology, future research will continue to focus on the social relationships that clubs need to consider with their fans as they largely return to pre-COVID-19 conditions (Lawrence and Crawford, 2022). In addition, it will be interesting to see how the new context and paradigm of the pandemic generate novel research ideas in the areas of gender, sexuality, and ideology.

#### 5.1.3. Business

Business research in soccer changed significantly during the pandemic compared to before it. Only the topic keyword cluster related to determinants of the market value of professional soccer clubs was similar in both periods (Kalen et al., 2019; Kirschstein and Liebscher, 2019; Wheatcroft, 2020; Gyimesi and Kehl, 2021). We found two related topics in the pre-COVID-19 period and one during the pandemic period. Timely research topic keywords exploring changes in key market value determinants, such as economic issues for clubs due to lockdowns, league reductions, and fan management, were also found in the pre-COVID-19 period (Herold et al., 2021; Quansah et al., 2021). In other words, active research approaches and practices are evident in both periods, as factors affecting market value and strategies to address them must continue to evolve in response to the changing environment.

In the pre-COVID-19 period, several business-focused keywords related to soccer were found in the topics. Research topics inferred from keywords such as “spectator attendance determinants,” “brand image,” “sponsorship,” and “loyalty” are typical of business research and are expected to continue being explored in the post-COVID-19 era, just as they were in the pre-COVID-19 era (Wang et al., 2018; Gyimesi, 2020; Pradhan et al., 2020; Valenti et al., 2020; Skinner and Smith, 2021). During the COVID-19 pandemic, many of the typical business-related keywords that were common in the pre-COVID-19 period disappeared. In addition, there were fewer keywords and topic clusters that can be identified as distinct business research topics compared to before the pandemic. The only business-related topics we found consisted of keywords related to CSR and CRM strategies, which are highly relevant to the socio-business research covered in the Sociology section (Fiadotava, 2021; Radmann and Karlen, 2022).

In fact, it could be inferred from the topic modeling results that soccer business research during the COVID-19 pandemic assumed a relational approach to preventing the loss of fans, which loss was predicted to be catastrophic due to the physical void caused by COVID-19. During the pandemic, soccer businesses tried to maintain and rebuild relationships with fans as potential consumers to prevent catastrophic losses due to physical voids such as lockdowns, unattended games, and shrinking consumption (Brand et al., 2023; Fenton et al., 2023). The topic keywords and clusters suggest that many strategies for fostering social relationships to overcome this crisis have been attempted in the interdisciplinary research of socio-business.

Furthermore, the topic modeling results demonstrate that the keyword clusters generally differ between both periods. The change in keyword clusters does not imply a change in research topics but rather a difference in the amount of research that was actively conducted at different times. The keyword clusters and topics found in both periods are those that have been studied in the soccer business field from the past to the present. Lee et al. (2022) reported that new business paradigms are emerging in the field to cope with the economic crisis of sporting organizations that have experienced fractured player–club and fan relationships and near bankruptcy. These new strategies and efforts to overcome the crisis will have their own set of failures and successes, and the role of football business research in this process needs to be explored specifically.

### 5.2. Sports science and sports medicine in soccer

#### 5.2.1. Technology

Technology research in soccer was divided into independent Topic Areas in both periods. In the pre-COVID-19 period, various types of research were conducted. Research on soccer match analysis has been ongoing since the late 1990s when the importance of match analysis was raised, for example, regarding the development and application of technology for more sophisticated analysis or proposing analysis results from different perspectives using recorded items utilized for specific match analysis factors (Delibas et al., 2019; Stein et al., 2019; Wu et al., 2019; Sheng et al., 2021). In addition, research related to stadium construction based on construction engineering to overcome environmental factors was a topic that confirms the scope of science and technology in soccer (Llarena et al., 2018; Bonser et al., 2020). During the COVID-19 pandemic, the clusters in the topic area were reduced, and only one topic was clustered with keywords that had different meanings than before the pandemic. The only independent cluster of keywords in the technology topic area during the pandemic was found in the soccer area that discussed advanced AI movement-tracking technology (Jin, 2022; Zou et al., 2022).

Similarly, we found studies from both periods regarding the development of individual technologies such as image processing and humanoids to improve the AI performance of soccer robots (Bai et al., 2020; Hu et al., 2021; Janos et al., 2022). The soccer robot is more of a source technology for maximizing robot and AI technology than a technology applied to the actual soccer field (Park et al., 1997; Samani et al., 2004). However, although the active participation in and performance of technology research may appear somewhat reduced during the COVID-19 period, the level of technology for collecting and analyzing game data (numbers, images, videos, etc.) was actually growing rapidly (Almulla et al., 2020; Kelly et al., 2020). Considering past experiences when humanity has developed science and technology to overcome crises, Garfin (2020) predicted that the pandemic period would also see remarkable changes. Therefore, it is expected that AI, robotics, immersive virtual technology, metaverse, blockchain, and all-encompassing WEB 3.0 technologies will be applied to the field of soccer in the process of turning the current crisis into an opportunity (Hotaran et al., 2022; Lorabi, 2022; Nobari et al., 2022). In this process, soccer technology research will continue to be an independent field of study.

#### 5.2.2. Soccer performance

In soccer, as in all sports disciplines, the search for new processes to improve player performance has been the focus of many experts and researchers. Research on soccer player performance has been actively conducted in soccer research, including exploring various determinants of performance ability and seeking effective interventions (Forcher et al., 2022). In other words, we found soccer performance keywords and topics about improving the measurement and ability of individual physical and athletic performance factors to be the most extensive, forming large and small clusters (Modric et al., 2019; Altmann et al., 2021; Maciejczyk et al., 2021).

For example, soccer performance research in the pre-COVID-19 period explored the relationship between performance-determining factors such as BMI and athletic performance related to sprinting (e.g., distance, speed, and acceleration) and soccer performance in actual matches (Rivilla-Garcia et al., 2019; Maciejczyk et al., 2021; Fernandez-Galvan et al., 2022). In addition to physical factors, performance factors focused on athleticism such as change of direction (COD) and reactive agility were explored (Little and Williams, 2005; Haugen et al., 2014; Carling et al., 2015). Furthermore, performance studies focused on cardiovascular aspects such as heart rate and cardiac output in match and training situations, while psycho-physiological studies measured the association between hormonal and psychological states during athletic performance (Buchheit et al., 2012; Faina et al., 2013). On the other hand, factors directly linked to winning on the field have undeniably been central to soccer performance research, with keyword clusters exploring the relationship between individual performance and the kinesiology expected of soccer performance, including motor behavior (Pain and Harwood, 2007; Abdullah et al., 2016; Williams, 2020).

Regarding the period during the COVID-19 pandemic, we found several similar types of studies to those published before the pandemic. Except for studies exploring goalkeeper behavioral traits and soccer players’ sleep quality, habits, and performance, performance studies focusing on kicking and measurements via GPS had mostly similar keyword compositions (Goes et al., 2021; Oliva-Lozano et al., 2021; Pons et al., 2021; Keemss et al., 2022; Vu et al., 2022). However, a more detailed observation suggests that soccer performance studies related to athleticism and physical level, which require direct performance measurement, decreased from the clusters and keyword compositions. It is difficult to conclude that these studies have disappeared by monitoring only the topic composition. However, the environment of changed lockdowns and league schedules, not having spectators, etc., during the COVID-19 pandemic inevitably made it difficult to measure or develop the physical and athletic performance factors that most closely relate to the actual game (Sekulic et al., 2021; Radziminski et al., 2022).

Nevertheless, the relationship between diet and performance and approaches to talent discovery and skill development in elite soccer players has been a consistent theme throughout the years (Braun et al., 2018; Ksiazek et al., 2020; Carter et al., 2021; McGuire et al., 2023). During the COVID-19 pandemic, the relationship between the maturation of basic fitness and athletic performance indices and soccer performance was studied from a variety of perspectives, which could be inferred to be driven by the field and the academic need to identify and develop talented players for team development despite the chaotic environment.

#### 5.2.3. Performance training

Performance training topics that emerged during the COVID-19 pandemic included jump training effectiveness studies validating improvements in various performance components, the link between stretching and performance, and training-induced improvements in muscular and aerobic capacity, as well as several interventions that are specific to improving performance (Bishop et al., 2021a, b; Huang et al., 2022; Nunez et al., 2022; Parpa and Michaelides, 2022). The small-sided game (SSG), the closest simulation to soccer practice, and the concept of session ratings of perceived exertion to reduce the difference between a player’s perceived load and physiologically measured load are more direct efforts to create effective training volume and training strategies (Dello Iacono et al., 2021). Research topics on minimizing overload and planning effective training processes for peak performance during the season are similar to those in the area of soccer performance in the pre-COVID-19 period.

All competitive sports require planned, controlled training to achieve high performance and improve team competitiveness (Mujika, 2017). Soccer is no exception, and many studies have proposed and validated proactive training programs (Randers et al., 2010). However, among the pre-COVID-19 topics, it is difficult to find a clear identity for the keyword “training” or a training area, because soccer performance and performance training were not separated into distinct topic areas and clusters. Similarly, in training sessions that require different physical abilities, there is a lot of overlap in concepts as researchers try to review a player’s athleticism, tactical understanding, physical development, etc., and compare them to actual competitive matches (Altmann et al., 2021; Teixeira et al., 2021). Therefore, it could be interpreted that the concepts were extracted as the same word in the NLP process, leading to the sharing of keywords between soccer performance and performance training. However, training is strictly differentiated from a match because it is based on a rational and controlled environment intended to uniformly equip a player with various performance factors (Fuller et al., 2006).

The reduced opportunities for actual matches due to lockdowns and the reduction in matches during the pandemic suggest that performance training in soccer has become relatively more prominent as an external factor. During the COVID-19 pandemic, many football organizations had to be adaptable and flexible in the way they trained their players (Bisciotti et al., 2020). In a strange, high-risk environment, players and coaches have had to be proactive in adapting training programs and schedules to changing restrictions and guidelines, and in some cases, switching to training that can deliver maximum impact in a short time (Jukic et al., 2020). In this process, many of the concerns of coaches and athletes would have been inevitably projected onto research, and some of these research attempts have emerged as topic areas.

However, no keywords were found to recognize the application of remote training methods and their underlying technologies, including virtual reality, AI, and wearables, which were used to a large extent during the pandemic. The time required to develop new approaches to respond to extraordinary change, including how long it takes for technologies to be fielded and studied and results to be interpreted, applied, and published, makes us hopeful for creative research trends in the future. In addition, new research topics that can be explored include the comparison of face-to-face training, as identified in this study, with newly published, technology-based training programs and the effectiveness of hybrid training programs that incorporate both elements.

#### 5.2.4. Injury: lower limb

Two injury-related topics emerged in the pre-COVID-19 period, consisting of keywords for specific injury sites in the lower extremities and factors that lead to injury. In soccer research on injuries in general, lower limb injuries have the highest prevalence (Zuke et al., 2018; Al Attar and Alshehri, 2019), but the pre-COVID-19 topics regarding the head, brain, and other body parts were clustered within various topic areas, and the lower limbs are no exception. In this regard, the most common on-field injury for male professional players is a hamstring tear; for female players, it is the knee, which has received considerably less academic attention than other injuries (DeLang et al., 2019; Baroni et al., 2020; Owoeye et al., 2020).

However, during the COVID-19 pandemic, lower-injury research has seen an increase in topics and a more granular focus, including studies of specific injury triggers and interventions for recovery. The injury sites studied were the hip, pelvis, hamstring, ankle, and knee, and the research covered a variety of topics, including injury triggers, repetitive injuries to the same site, injury prevention, and rehabilitation (Fernandez-Baeza et al., 2022; Flore et al., 2022; Mendez-Villanueva et al., 2022; Mohammad and Elsais, 2022; Roughead et al., 2022).

Several reasons have been speculated for the change in research topics related to head and lower extremity injuries during COVID-19 as opposed to pre-COVID-19. Shibukawa and Hoshikawa (2022) limited their study to the Japanese soccer league but demonstrated that heading injuries decreased after the implementation of the revised FIFA rules in 2019. In addition, coaches have been issuing instructions in training to discourage heading (Muller and Zentgraf, 2021; Waring et al., 2022). On the other hand, studies of soccer injuries during the pandemic did not report a change in overall injury rates, but there were reports of an increase in lower limb injuries, including hamstrings, in professional matches that resumed after the lockdown period because players were not ready to play (Orhant et al., 2021; Seshadri et al., 2021; Thron et al., 2021; Mazza et al., 2022; Thron et al., 2022).

It could be assumed that there would inevitably be more training situations than matches during the pandemic and that research based on training environments would be more active. Even in pre-COVID-19 practical match situations, dynamic headers that could cause injury were discouraged to the extent that regulations were implemented to minimize head injuries (Yang and Baugh, 2016; Quintero et al., 2020), and during the lockdowns, more training situations were experienced than games (Bisciotti et al., 2020; Washif et al., 2023). In training, a player rarely makes the same headers as they would in a real game, but they still must work their lower extremities. Small-sided games, one of the soccer training methods, include smaller-than-standard goals, so there are relatively fewer dangerous contacts, especially headers, that often occur in real matches, and some small-sided games even restrict header play (Owen et al., 2014).

These different causes may have contributed to the increased representation of lower limb injury research in the topic modeling results over time. More importantly, however, the problem of functional decline and injury in the lower extremities due to reduced training and playing opportunities has been reported in much of the current research on lower extremity injuries (Rampinini et al., 2021; Paravlic et al., 2022). Demonstrating different interventions to address these injuries might be the most urgent future research direction.

#### 5.2.5. Injury: head and brain

In contrast to the lower limbs, in the pre-COVID-19 era, head injury research was divided into at least two topic areas. One topic area attempted to explore the relationship between impact size and injury by measuring the impact of a header (e.g., physical measurements, survey measurements, etc.) (Campolettano and Rowson, 2021; Miller et al., 2021). The other topic area addressed brain injury in soccer players, with a focus on pathological brain injuries such as encephalopathy, dementia, Alzheimer’s, and ADHD (Rutherford et al., 2019; Didehbani et al., 2020; Reyes et al., 2020). In soccer, head injuries cause such serious sequelae that evidence-based rules and policies have been proposed and headers controlled in matches and training (Beaudouin et al., 2021; Chandran et al., 2021; Haarbauer-Krupa et al., 2021; Ye et al., 2022). In addition, some studies have highlighted reports of sequelae focusing on neurocognitive symptoms after brain injury (Sandmo et al., 2020; Wahlquist and Kaminski, 2021). The seriousness of the problem is evidenced by several research approaches that explore the various factors that influence head and neck injuries, as well as policy and institutional limitations to prevent such injuries.

One cluster of topics emerged during the pandemic that addressed brain injuries, including concussions. Because of the reduced number of matches and the lockdowns during the COVID-19 pandemic, the head injury topic might have been underrepresented more dramatically than the overall physical injury topic. While there were many factors contributing to this relative decline in topics, the variables of attrition and decreased frequency of research cannot be overlooked. As previously described, the relative contraction during the coronavirus period could be attributed to a relative reduction in live matches compared to previous periods, resulting in a decrease in exposure to dangerous head injuries, including headers, and an increase in lower limb injuries (Bamac et al., 2011; Rodrigues et al., 2019; Mazza et al., 2022).

However, Shibukawa and Hoshikawa (2022) reported a reduction in head injuries in some professional leagues following FIFA’s goal-kicking policy. Coincidentally, the pandemic started not long after this policy. At this point, it is difficult to prove whether this reduction was due to the policy or the pandemic. Currently, it is expected that reports of brain injuries and research to prove exact causations will follow.

#### 5.2.6. Other injury

In addition to the lower limbs and head, injuries in soccer occur in a variety of other areas, and research has been published on these injuries. Although not organized into independent topic areas, discrete clusters of topics identified thematic areas of injury research. Some topics consisted of many of the same keywords, regardless of the time period. First, studies exploring the association between bone density and mineral content and injury were published before and during COVID-19, with various studies focusing on bone composition, including differences in bone density and injury in soccer players according to gender and bone density and injury according to sport (Lozano-Berges et al., 2018; Gomez-Campos et al., 2019; Filippella et al., 2020; Bergamo et al., 2023).

Keyword clusters indicative of cardiac injuries in soccer players were also found in both periods. Soccer research on the heart can be categorized into studies examining soccer players’ heart disease and cardiac responses after matches and training (Martin-Sanchez et al., 2011; Unnithan et al., 2022). The topics in this study cover mild and severe heart-related injuries, including coronary artery disease and sudden cardiac death in soccer players. There are specific reports of a long history of cardiac research regarding soccer players from 1955 to the present (Higgins and Andino, 2013). There has also been a long history of exploration into the most potentially fatal of soccer injuries, including in-competition deaths and chronic heart disease in retired players.

In both periods, we also found studies that explored turf type as a factor, as opposed to specific injury sites. Natural and artificial turf comprise two of the many influential external environments in which players play (Nedelec et al., 2013; Ataabadi et al., 2017). Importantly, (Silva et al., 2017) reported that the interaction between a player’s style of play and the surface condition of the turf may be a contributing factor to injury. Multiple studies have been published on the relationship between turf type and injury prevalence, as well as literature reviews and meta-analyses, and to date, research continues to be attempted (Calloway et al., 2019)

Dietary habits, energy intake, and metabolism were always clustered as one or more themes in performance studies, but there was also a strong presence of studies demonstrating a link between these themes and injury (Cavarretta et al., 2018; Faltstrom et al., 2022). This cluster could be found in both periods, with the primary approach being the relationship to injury, which can be influenced by behaviors related to diet. Additionally, COVID-19 keywords were found in topics during the pandemic, suggesting that changes in dietary behavior under lockdowns during the pandemic have not been overlooked by researchers (Carter et al., 2021). To date, there are no published studies demonstrating a relationship between problematic dietary behaviors and injury prevalence resulting from lockdowns during COVID-19. However, now that the pandemic has ended and normalcy has returned, there is concern that poor dietary habits from past lockdowns may continue to lead to health problems and injuries (Haan et al., 2021; Rico-Gonzalez et al., 2021). The study of dietary habits is worthy of continued exploration until the effects of the pandemic have had time to dissipate.

In addition, the relationship between the soccer environment and injury, which is bound to have changed through the pandemic, is a timely topic. Topics describing the relationship between training load and performance, which were found in the performance research area during the pre-COVID-19 period, have been partially transformed into keyword clusters linking training load to injury (Bache-Mathiesen et al., 2022). The risk of injury is also inevitable because of the increased training time during the pandemic, and the cluster of topics related to injury experiences and concerns may also reflect the temporal nature of the pandemic (Schuttler et al., 2022). In addition, clusters containing keywords about returning from injury and athletes returning after the pandemic are not conclusions based on experiments or surveys but rather topics that can be expected to be the subject of further research (Ross et al., 2021; Maestro et al., 2022).

On the other hand, the psychosocial and medical issues of soccer players and other practitioners who had to face restrictions not only in matches but also in training and daily life due to league suspensions, curtailments, and lockdowns (Balyan et al., 2021; Karagun et al., 2021; Papagiannis et al., 2022) that were unavoidable during the COVID-19 pandemic period suggest a research approach of qualitative and quantitative exploration to identify these issues in the present and prepare for the threat of recurrence in the future.

## 6. Limitation

In the present research, we utilized the Web of Science (WOS) database to carry out a topic modeling analysis, encompassing articles indexed in the Science Citation Index Expanded, the Social Sciences Citation Index, and the Arts & Humanities Citation Index. We anticipate that a more integrated approach in data collection and analysis could be achieved by incorporating diverse academic databases such as Scopus and SportDISCUS, alongside WOS. Furthermore, it is pertinent to highlight the potential role of the BERT model, which forms the foundation of BERTopic, in enhancing the quality and depth of analysis. BERT, known for its capability to acquire intensive domain knowledge in diverse fields, can facilitate the extraction of more pertinent keywords and topics when fine-tuned with specialized domain knowledge. Future research endeavors could explore the outcomes of employing a BERT model that has been fine-tuned using sports or soccer-centric documents. Lastly, as the global society transitions into the post-pandemic period, it would be significant to adopt a longitudinal perspective in examining whether the trends in soccer research are reverting to the patterns observed before the advent of the COVID-19 pandemic.

## 7. Conclusion

The COVID-19 pandemic has had a clear impact on soccer research trends. Topic modeling revealed a context in which the pandemic led to changes in research priorities that reflect changes in soccer participation, training routines, and even the sports business environment. First, the interdisciplinary nature of topics across both time periods emerged, spanning psychology, sociology, business, and technology. This emphasizes that socioeconomic phenomena are emerging from soccer that are relevant to a variety of academic disciplines. On the business side, concerns about the economic impact of the COVID 19 pandemic were reflected in the research. It also emphasized the importance of team-fan and player-fan relationships to overcome this crisis. Second, before and during the pandemic, sports, including soccer, still valued performance. In both periods, there was a strong emphasis on training, an expected theme given soccer players’ constant efforts to improve their performance. However, during the COVID-19 pandemic, a shift in themes revealed a focus on developing fast-paced, highly efficient training sessions in response to various constraints. Third, this change in the soccer environment during the pandemic was inferred as the reason for the shift in research focus from head injuries to lower limb injuries. It could be that training routines changed as match play was restricted, which affected the types of injuries players faced, or it could be that the pandemic led to less physical contact during matches, which led to fewer head-related injuries. This shift in focus could be a direct result of the pandemic, but it could also be due to new rules, advances in technology, or a shift in awareness of the issue of injury sites. Furthermore, the relative decline in head and brain injury research during the pandemic does not necessarily mean that the issue has become less important; rather, it may be an opportunity for researchers to explore the long-term effects of these injuries and the potential benefits of reducing exposure to dangerous situations. In conclusion, while the COVID-19 pandemic has clearly impacted soccer research trends, the overall direction of research on player performance, injury prevention, and technology integration remains macroscopically consistent compared to pre-COVID-19. However, as the world adapts to the new normal, soccer research must evolve to optimize athlete health and training methods and continue to advance performance. Future research must bridge these gaps and develop a holistic strategy to meet the ever-evolving challenges of this popular sport.

## Data availability statement

The original contributions presented in the study are included in the article/supplementary materials, further inquiries can be directed to the corresponding author.

## Author contributions

JL and DH contributed to the conception and design of this study. SS collected the database. YK performed the topic modeling analyses. JL, S-BP, and DH wrote sections of the manuscript, contributed to manuscript revision, read, and approved the submitted version. All authors contributed to the article and approved the submitted version.
